# Interface strategies in monolingual and end-state L2 Spanish grammars are not that different

**DOI:** 10.3389/fpsyg.2014.01525

**Published:** 2015-01-13

**Authors:** María C. Parafita Couto, Virginia C. Mueller Gathercole, Hans Stadthagen-González

**Affiliations:** ^1^Leiden University Center for Linguistics and Leiden Institute for Brain and Cognition, Leiden UniversityLeiden, Netherlands; ^2^Linguistics Program, English Department, Florida International UniversityMiami, FL, USA; ^3^Department of Psychology, University of Southern MississippiHattiesburg, MS, USA

**Keywords:** interfaces, focus, unaccusative, unergative, L2 acquisition, Spanish

## Abstract

This study explores syntactic, pragmatic, and lexical influences on adherence to SV and VS orders in native and fluent L2 speakers of Spanish. A judgment task examined 20 native monolingual and 20 longstanding L2 bilingual Spanish speakers' acceptance of SV and VS structures. Seventy-six distinct verbs were tested under a combination of syntactic and pragmatic constraints. Our findings challenge the hypothesis that internal interfaces are acquired more easily than external interfaces (Sorace, 2005, 2011; Sorace and Filiaci, 2006; White, 2006). Additional findings are that (a) bilinguals' judgments are less firm overall than monolinguals' (i.e., monolinguals are more likely to give extreme “yes” or “no” judgments) and (b) individual verbs do not necessarily behave as predicted under standard definitions of unaccusatives and unergatives. Correlations of the patterns found in the data with verb frequencies suggest that usage-based accounts of grammatical knowledge could help provide insight into speakers' knowledge of these constructs.

## Introduction

This article concerns the extent to which high-functioning L2 Spanish speakers have acquired the full grammar for the expression of focus. In particular, we look at the language-specific means of expressing linguistic focus within the bilingual's two grammars, Spanish and English. Linguistic focus concerns that portion of a sentence that contributes the most relevant new information, the non-presupposed information, of the utterance. As such, focus stands at the interface between syntax, phonology, and pragmatics, as what is focused in a sentence, expressed syntactically and/or phonologically, depends directly on the discourse and pragmatic intent in which the sentence is embedded. The main questions explored in this article are (i) whether bilinguals' grammars converge with (or diverge from) those of monolinguals and (ii) whether certain linguistic areas are more vulnerable to influence than others. We will particularly examine the expression of focus in Spanish through the syntactic operations of word order. We test both Spanish monolinguals and Spanish-English functional bilinguals.

Recent research in linguistic theory has focused on the properties that (external) interface conditions impose on the design of the language faculty (Chomsky, 2005), since the output of the computational system has to be interpreted by other cognitive systems (sensory-motor systems and conceptual-intentional systems). L2 research has recently posed the question of how well L2 learners are able to integrate linguistic phenomena pertaining to interfaces (White, 2009). Sorace (2005), Sorace and Filiaci (2006) and Tsimpli and Sorace (2006) have formulated the interface hypothesis, which argues that phenomena contained within narrow syntax or lying at internal interfaces can be completely acquired in the L2, whereas full acquisition may not be possible for phenomena placed at external interfaces. These authors claim that narrow syntax is not a problem for acquisition while internal interfaces (at least syntax/semantics) are argued to be relatively unproblematic. However, external interfaces (e.g. syntax/discourse) are claimed to be a locus of instability in bilingual speakers. More recently, Sorace (2011) emphasized that the interface hypothesis predicts that both syntactic and pragmatic conditions are acquirable but the integration of both conditions remains less than optimally efficient, giving rise to optionality.

In this paper we explore a phenomenon that lies at both the external and internal interfaces: the expression of focus (syntax-discourse interface) in sentences with intransitive (unaccusative and unergative) verbs (syntax-semantics interface). We do this by exploring whether Spanish-English functional bilinguals have problems in coordinating the syntax and the pragmatics in focus contexts through the distribution of subject-verb (SV) and verb-subject (VS) word order. Spanish has flexible word order, while English is more rigid. While in neutral focus contexts SV is the canonical word order in Spanish, VS order can result from different kinds of syntactic operations (Lozano, 2003). According to Contreras (1978), Suñer (1982) and Zubizarreta (1998), there is a clear tendency for speakers to produce VS order for unaccusative verbs, and the most common discourse-neutral order for unergatives is SV. The intransitive verb class is of interest because of the contrast between Spanish and English. English allows stress on the preverbal subject for both unaccusative and unergative verbs in “out of the blue” contexts, in which the subject is the focus (e.g., “A book fell,” cf. Schmerling, 1976; Selkirk, 1984; Nava, 2007). In the same context in Spanish, the subject would occur in post-verbal position and stress would fall on the rightmost constituent (“Se cayó un libro”).

This paper examines Spanish-English bilinguals' knowledge of the Spanish forms. The paper is structured as follows: First, we summarize the effects of unaccusativity and focus on word order in Spanish, report on previous research on the acquisition of Spanish word order patterns, and present the research questions and hypotheses. We then report on the experimental evidence bearing on these questions, followed by a discussion of our findings in relation to the perspective of previous research.

## Theoretical background: is word order at the interfaces acquirable?

### Research question

Spanish and English are both SVO languages, but VS is also possible in both languages:

(1) Llegaron los niños.
      Arrived the kids
     “The kids arrived”
(2) Here comes the sun.

However, in Spanish post-verbal subjects seem to be produced freely with all verb classes:

(3) Ha telefoneado María al presidente. (transitive)
      has phoned Mary the president
      “Mary has phoned the president.”
(4) Ha hablado Juan. (unergative)
      has spoken Juan
      “Juan has spoken.”

(5) Ha llegado Juan (unaccusative)
      has arrived Juan
      “Juan has arrived.”

In Spanish, inversion is usually a means of “focalization”: preverbal subjects are topics (given information) and post-verbal subjects are focus (new information) (Zubizarreta, 1998; Belletti, 2001, 2004). e.g.,

(6) ¿‘Quién ha llegado/hablado?
      Who has arrived/spoken?

i. Ha llegado/hablado Juan
ii. #Juan ha llegado/hablado

In neutral (non-focus) contexts, subjects tend to be discourse-initial, except in the case of unaccusative verbs:

(7) a. Una mujer gritó (unerg)
      b. # Gritó una mujer.
      ‘A woman shouted.’
(8) a. # Una mujer llegó. (unacc)
      b. Llegó una mujer.
      ‘A woman arrived.’

Previous studies on Spanish native speakers show that verb choice may determine word order (Pinto, 1999; Hertel, 2003; Lozano, 2003, 2006a,b). Default word order is reported to be SV for unergatives and VS for unaccusatives (i.e., determined by the lexicon-syntax interface). Word order in focused contexts is VS for both verb types (i.e., determined by the syntax-discourse interface).

#### Unaccusativity: syntax or semantics?

Baker (1983) remarked that “all seemingly intransitive verbs are not created equal” (p. 1). According to the Unaccusative Hypothesis, there are two classes of intransitive verbs: unaccusatives and unergatives (Perlmutter, 1978; Perlmutter and Postal, 1984). For some researchers, the difference between the two types is semantic; for others, it is syntactic. Semantically, the two types of verb differ in that, whereas the subject of an unergative verb actively initiates or is actively responsible for the action expressed in the verb, the subject of an unaccusative verb does not. Subjects of unaccusatives bear the semantic role of theme or patient, usually associated with the objects of verbs. In Dowty's (1991) and van Valin's (1999) terms, the difference between the two classes of verbs reduces to differences in agentivity and telicity. Unergative verbs are typically agentive and denote an atelic process (run, walk, work), while unaccusative verbs (die, disappear, exist) are non-agentive and telic, usually denoting a change of some sort.

In contrast, for generative linguists such as Burzio (1986) and Rosen (1984) the distinction between the two classes of verbs is mainly syntactic. According to the Unaccusative Hypothesis, the single argument of unaccusatives is syntactically a direct object, while the single argument of unergatives is the subject. Thus, although superficially the sentences “The leaf fell” and “The bird chirped” both show NP-V word order, the former involves NP-movement from object to subject position (9), while in the latter the NP is base-generated in subject position (10).

(9) The leaf_i_ fell t_i_.
(10) The bird chirped.
                                     (Friedmann et al., 2008)

Even though Burzio's (1986) formulation of the Unaccusative Hypothesis has been widely accepted, it is not uncontroversial. For example, Rappaport Hovav and Levin (2001:792) present counter-examples to syntactic accounts of English resultatives which are based on the assumption that result XPs are predicated of underlying direct objects. They concluded that “Our work calls even more seriously into question the existence of any evidence for the syntactic encoding of unaccusativity in English.”

**Figure 1:**
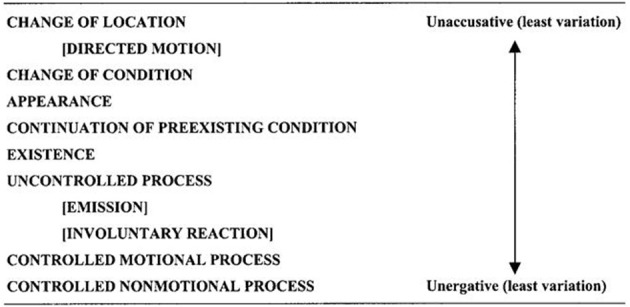
**Sorace's unaccusativity hierarchy (Sorace and Shomura, 2001)**.

These different approaches have led to the result that in the literature, unaccusatives are not consistently classified in semantic or syntactic terms. Hatcher (1956) offered the first semantic classification. De Miguel (1993) took into account both theta-role structure and the semantics of the verb. Building on Burzio, Sorace (1995, 2000) considered both syntactic and semantic aspects in her classification. She proposed that there is a universal continuum (a “hierarchy”) of gradients of unaccusative/unergative verbs, a continuum of potentially universal significance. This hierarchy is based on the semantic concepts of telicity and agentivity. The extremes of the continuum (“core”), with non-agentive, telic meanings on one end and agentive, non-telic on the other, correspond to the prototypical unaccusative and unergative verbs. The verbs in the middle are more or less unaccusative or unergative, depending on where they lie on the continuum. The types of semantic meanings that fall between the two extremes are as shown in Figure 1.

Crosslinguistically, unaccusative verbs fall on one end and unergative verbs on the other, and the two categories of verbs are distinguished by differences in syntactic behavior. Languages differ, however, in terms of the point at which unaccusatives are separated from unergatives along the hierarchy. But Sorace and Shomura (2001) raise the issue of the learnability of the unaccusative/unergative dichotomy and posit that the difficulty in acquiring this split intransitivity (unaccusatives vs. unergatives) stems from the problem of systematically linking “a multicategorial lexical-semantic level to a necessarily binary syntactic level…” (p. 249).

#### Unaccusativity and learnability

Montrul (2001) points out that for linguists and psycholinguists working within the generative framework (Chomsky, 1981, 1995), who assume that there is a syntactic difference between the two classes of intransitive verbs, the acquisition of unaccusativity represents a classic “poverty of the stimulus” problem. On the surface, all intransitive verbs look alike: they have one argument. How does the learner find out, solely from positive evidence, that these two verb classes have different underlying representations? Furthermore, when the learner finally finds out that there is a distinction, how does he/she classify newly acquired intransitive verbs? van Hout (1996) argues that the L1 learner already comes equipped with knowledge of the syntactic distinction (i.e., it is innate) but needs to find out which specific semantic notion is grammatically relevant for the unaccusative/unergative classification (telicity, change of state, transition, etc.).

Available studies report very few problems with the acquisition of intransitive verbs in L1 acquisition (e.g., van Hout et al., 1992 for Dutch). As argued by Montrul (2001), one of the main differences between child language acquisition and adults acquiring a second language is that second language (L2) learners already have a mature linguistic system in place. Therefore, if unaccusativity is universal, L2 learners presumably know about the unaccusative/unergative distinction (and know how it is expressed in their native language), although the semantic basis for the distinction might be different in the L1 and the L2. In English, certain unaccusative verbs can appear with existential subjects (“There appeared three men.”) and in the resultative construction (“The bag fell open.”) whereas unergative verbs cannot (“*There worked three men.” “*Mary laughed hoarse.”) (Perlmutter, 1978; Levin and Rappaport Hovav, 1995; Montrul, 2004). However, quite differently from what has been reported for L1 acquisition, a number of L2 acquisition studies have reported that unaccusative verbs, but not unergatives, cause problems for L2 learners of English and other languages and of various L1 backgrounds, especially at high intermediate and quite advanced levels (e.g., Yip, 1995; Oshita, 2001; Sorace and Shomura, 2001, inter multa al.). It has been reported that L2 learners have difficulty in determining the range of appropriate syntactic realizations of the distinction and that this difficulty can persist into near-native levels of proficiency (Hawkins, 2000).

Montrul (2005) rightly argued that Spanish is an interesting testing ground because, unlike Italian, which has auxiliary selection and *ne*-cliticization, Spanish does not provide such robust and clear syntactic and morphological evidence for unaccusativity. Furthermore, the topic has remained largely understudied in Spanish L2 acquisition. For example, Montrul points out that it is not known what role, if any, semantic subclass plays in the acquisition of these verbs. One very recent study (de Prada Pérez and Pascual y Cabo, 2012), however, suggests that even though Spanish heritage speakers use subject position differently in broad and narrow focus, they make no distinction between unergative and unaccusative predicates (contra the predictions of the Interface Hypothesis).

We will test below the assumption that unaccusativity corresponds to a syntactic phenomenon related to word order in Spanish and explore the possibility that semantics also plays a role in the classification of verbs, as has been indicated for other Romance languages (Sorace, 1993a,b, 1995). Before addressing these issues, however, it is essential to examine another factor that also constrains the distribution of SV/VS order in Spanish: Focus.

#### SV/VS: unaccusativity and/or focus

Lozano's (2003) dissertation was perhaps the first attempt to argue that the distribution of SV and VS in L2 Spanish is constrained both by universal principles like the Unaccusative Hypothesis and, at the same time, by discourse parameterizable features like presentational focus. He argues that learners' knowledge is convergent in unaccusative/unergative contexts (internal interface) yet divergent in presentational focus contexts (external interface). A few studies on the acquisition of the syntax-discourse interface previous to Lozano's dissertation had reported that presentationally focused subjects in final position are acquired late in L2 Spanish—e.g., Hertel (2003), as was also reported for L2 Italian (Belletti and Leonini, 2004). Ocampo (1990) and Camacho (1999) similarly reported that the acquisition of distinct word orders to mark focus in Spanish is acquired late or perhaps never in native-like fashion. More recently, Domínguez and Arche (2014) argued after looking at their native data that the linguistic evidence available for acquiring the syntactic properties of unergative and unaccusative verbs in Spanish is not completely transparent and that L2 speakers may not get clear evidence, which can explain why learners find the acquisition of SV–VS contrasts persistently difficult. However, they claim that their analysis is compatible with the view that L2 speakers eventually converge on the grammar of native speakers, and that this may well be the case with their advanced speakers as their experience in the L2 increases.

#### Research questions and hypotheses

In light of the contributions of syntax, semantics and information structure (i.e., Focus) to the acceptability and production of SV/VS word order in Spanish, the following research questions can be formulated with respect to L2 Spanish speakers who are long-standing functional^1^ bilinguals:

- Do long-standing functional Spanish-English bilinguals (L1 English, L2 Spanish) respect syntactic differences between unaccusative and unergative verbs?
- Does the hierarchy proposed by Sorace (2000) play a role in the acquisition/processing of these verbs in L2 Spanish?

In this study we set out to compare syntactic, pragmatic, and lexical influences on adherence to SV and VS orders in Spanish monolinguals and in fluent L2 speakers of Spanish. This study also looked for empirical evidence to test Beck's (1998) claim that optionality results in a permanent state even after long immersion in the language. We look at the distribution of SV/VS order in long-term Spanish L2 speakers and whether long experience with the L2 leads to convergent native-like behavior, as argued by Domínguez and Arche (2014). Currently, there is no consensus on the status of optionality in end-state grammars (Lozano, 2003, 2009). In addition, this study provides an empirical test for the interface hypothesis, which posits that internal interfaces (e.g., unaccusativity as a syntax-semantics interface) are not problematic for L2 acquisition while external interfaces (e.g., focus as a syntax-discourse interface) are problematic, acquired later, or never acquired. The following predictions were made:

– If syntactic knowledge of unaccusativity develops early, native speakers should have robust syntactic knowledge of the distinction between unaccusative and unergative verbs.
– If Sorace's Hierarchy is valid, we should observe differences in how monolinguals rate different semantic subclasses of unaccusative and unergative verbs.
– If there are differences between monolinguals and functional bilinguals, these should be observed in their ratings of the acceptability of unaccusative and unergative constructions.
– L2 speakers may have a less robust knowledge of the unaccusative/unergative distinction with non-prototypical verbs—i.e., verbs in the center of the continuum.
– If Lozano's claim regarding the ease with which L2 learners learn internal interfaces relative to learning external interfaces is correct, functional bilinguals should differ in their ratings from monolinguals more with regard to focus environments than with regard to verb class, given that focus lies at an external interface.

## The experiment

### Methods

We conducted an experimental study to test the knowledge of Spanish-English functional bilinguals in comparison with Spanish monolinguals with respect to unergative and unaccusative SV/VS alternations both in neutral and presentational focus contexts. For our study, we followed the methodology developed by Lozano (2003) but modified it to test a higher number [*n* = 76] of Spanish verbs classified according to Sorace's Unaccusative hierarchy.

#### Participants

A total of 40 subjects participated in the study. All but two of the participants lived in Madrid, Spain. A group of 20 Spanish monolingual native speakers (mean age 23, range 19–31) served as a baseline to compare with the L2 Spanish speakers' results. The experimental group consisted of 20 English native speakers aged 29–72 (mean age 46.6), eighteen of whom lived in Madrid, Spain, and had lived there for an average period of 20.7 years (range 3–47 years). The remaining two of the L2 Spanish speakers had lived for periods in Spain; one of them was a university psychology professor who had been married to a Spaniard for over 20 years, and the language of conversation in their home between themselves and with their children was Spanish, and the other was a college professor of Spanish literature who had been traveling to Spain on a regular basis since 1968 and used Spanish in her work environment on a daily basis. The onset of Spanish for the L2 bilinguals occurred between the ages of 16 and 38 (average age of onset: 21.4 years), and all speak Spanish on a daily basis at work and with their families. Three of the bilinguals were male, 16 female; 7 of the monolinguals were male, 13 female. The educational backgrounds of the bilingual participants was high school level or higher (4 high school, 12 BA/BSc or equivalent, 3 MA, PhD or equivalent). On a scale of 1 to 4 (1 = only some words, 2 = confident in basic conversations, 3 = fairly confident in extended conversations, 4 = confident in extended conversations), the bilinguals rated their English abilities at 3.95 (19 ratings of “4,” 1 of “3”) and their Spanish abilities at 3.74 (5 ratings of “3,” 15 of “4”). All participants signed consent forms and were paid for their participation.

#### Stimuli

**Figure 2:**
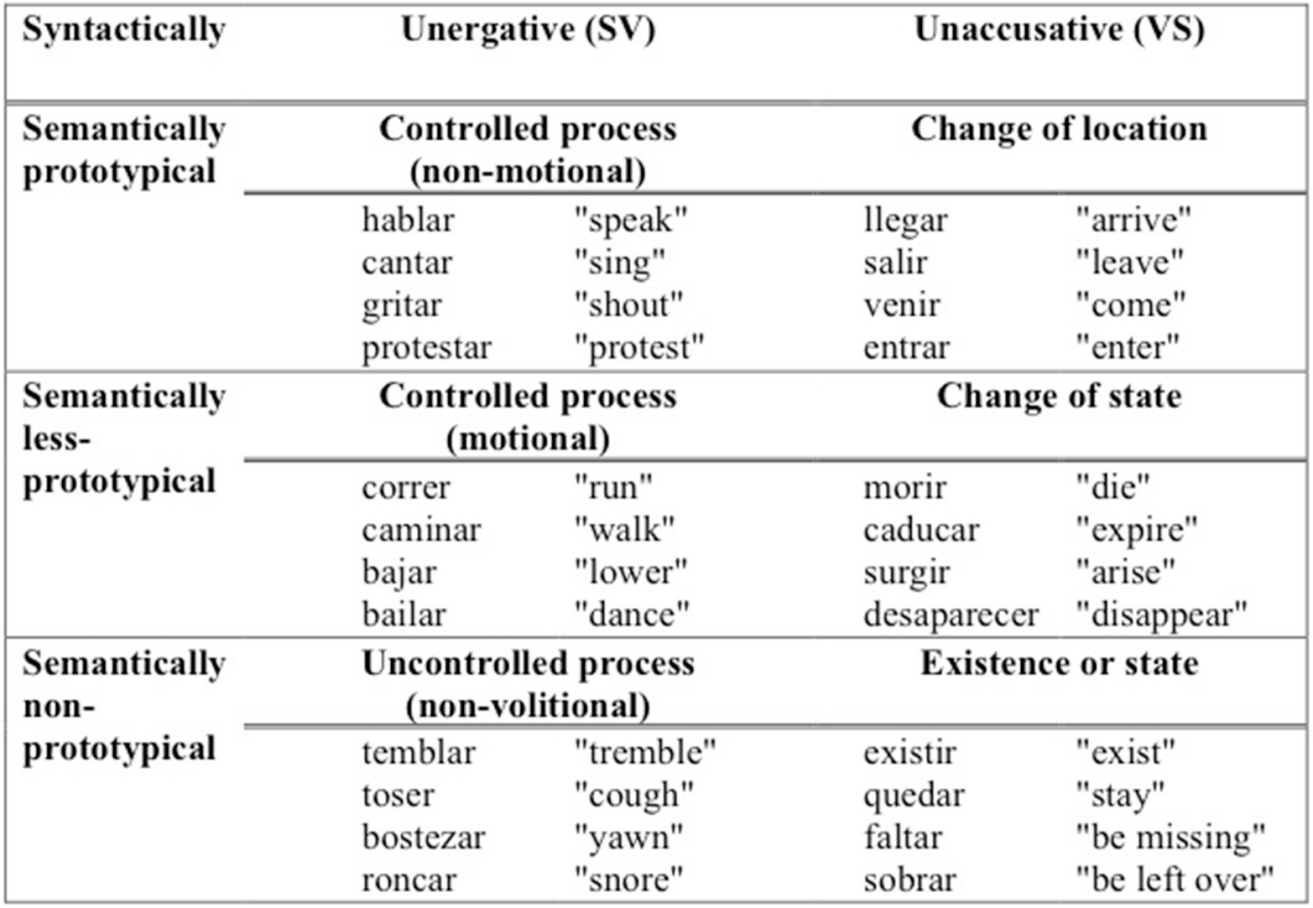
**Sample verbs**.

The instrument employed was an acceptability judgment test. Sentences were constructed with 76 distinct verbs embedded within short scenarios depicting a context of utterance. The target stimuli involved 19 unergative verbs in neutral contexts, 19 unaccusatives in neutral contexts, 19 unergatives in focused contexts, and 19 unaccusatives in focused contexts. The verbs tested included semantically prototypical, semantically non-prototypical, and semantically intermediate/less prototypical verbs, according to Sorace's Hierarchy. The verbs also were grouped according to whether they typically occurred with or without multifunctional se. Sample verbs are shown in Figure 2.

**Figure 3:**
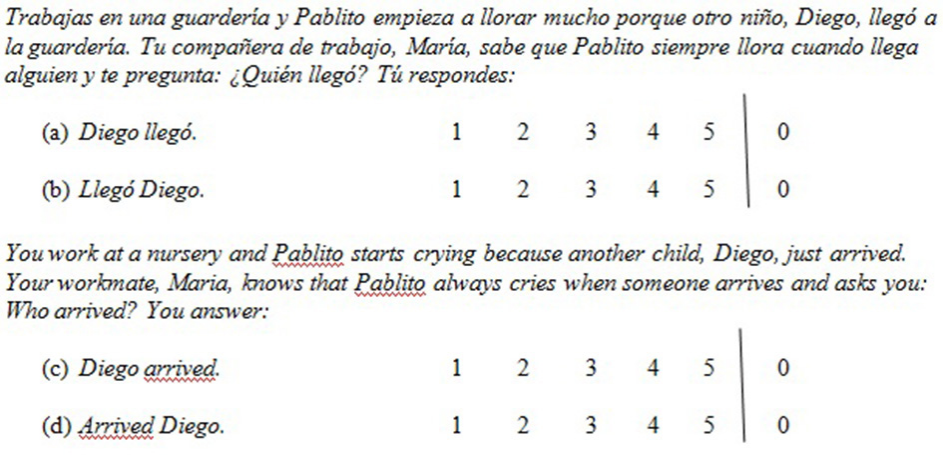
**Sample stimulus**.

Half of the conditions presented contexts appropriate for focused subjects and half for non-focused subjects. Each contextual setting ended with a question, followed by two possible replies (see Figure 3). The two possible replies represented different word orders (SV vs. VS).

**Figure 4:**
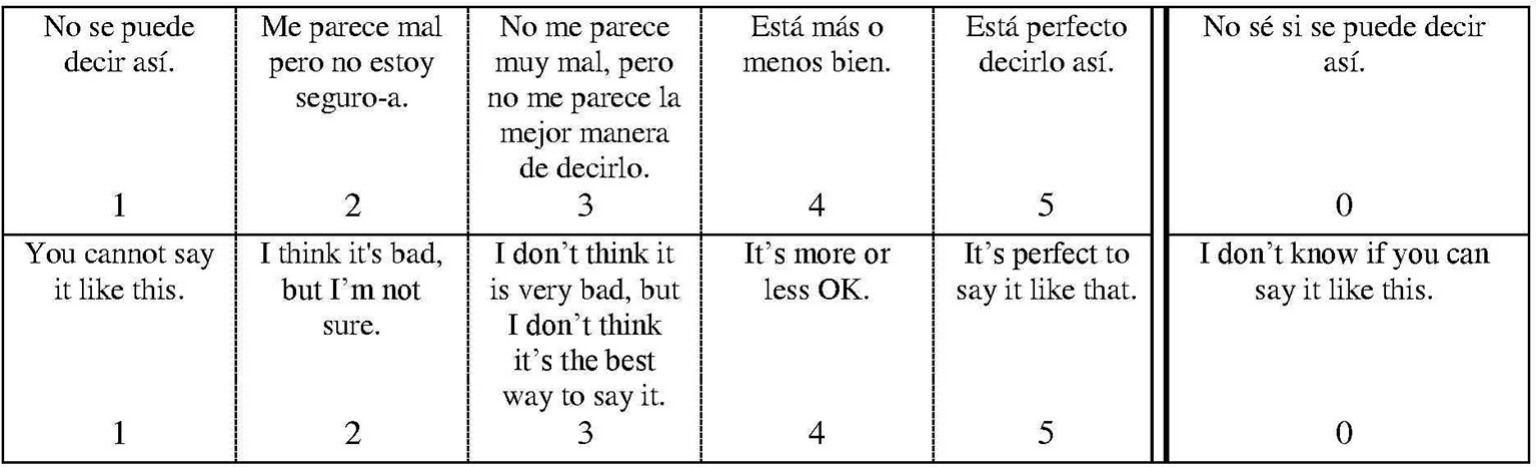
**Rating scale**.

As exemplified in Figure 3, each target sentence was accompanied by a 5-point Likert rating scale (see Figure 4). (Participants were given only the Spanish; the translation is provided here for the convenience of the reader.) Value 1 corresponded to *No se puede decir así* “you cannot say it like this,” value 5 corresponded to *Está perfecto decirlo así* “it's perfect to say it like that,” with values 2–4 several levels between these two extremes. (Value 0 corresponded to *No sé si se puede decir así* “I don't know if you can say it like this”).

Twenty-four control sentences were also included. These sentences involved pro-drop, with the two choice answers differing in the presence/absence of overt subjects. Two training stimuli were placed at the beginning of the test. These consisted of structures not related to those of interest here, one trial involving the position of a clitic pre- or post-verbally and the other the order of a noun-adjective sequence.

Four randomized versions of the test were created with the same sentences but with different sequential order. The sequential order was randomized following Cowart (1997) “blocking” procedure.

#### Procedure

Participants were asked to judge the acceptability of both sentences given. Following Lozano's model (Lozano, 2003, 2006a^2^), participants were given written instructions at the beginning of the test. The instructions highlighted that the researchers were interested in the participant's opinion of a set of sentences, as follows:

“El objetivo de este test es averiguar cómo te suenan ciertas oraciones en español. Es importante resaltar que sólo nos interesa TU opinión sobre ellas, es decir, si te parecen más o menos aceptables. El test no será corregido, sino que su finalidad es averiguar si ciertas oraciones suenan mejor o peor a los hablantes nativos de español.”

English translation (provided here for the convenience of the reader; the English version was not given to the participants):

“The objective of this test is to see how certain sentences sound to you in Spanish. It is important to stress that we are only interested in YOUR opinion about them—that is, if they seem more or less acceptable to you. This test will not be graded, but the goal is to see if certain sentences sound better or worse to those who speak Spanish natively.”

It also contained explicit instructions on how to complete the test and it detailed what the value scale meant, providing some examples, as follows:

“En cada número que sigue, verás una lectura corta. Léela primero. Luego le siguen dos oraciones muy parecidas. Oración (a) y oración (b). Queremos que juzgues, dada la lectura que acabas de leer, cómo suena cada oración. Cada una de las oraciones está seguida de la siguiente escala para puntuar cada oración:

**Table:** 

No se Puede decir así	Me parece mal pero no estoy seguro-a	No me parece muy mal, pero no me parece la mejor manera de decirlo	Está más menos bien	Est'a perfecto decirlo as'i	No sé si se puede decir así
1	2	3	4	5	6

Aquí te ponemos un ejemplo:
A ti siempre te gustaron mucho los churros con chocolate. Cuando eras pequeño-a, siempre que veías churros, le decías a tu madre:
  (a) Quiero comerlos. 1 2 3 ➄ 0
  (b) Los quiero comer. 1 2 3 ➄ 0

English translation (given here for the reader):

For every item below, you will see a short reading. Read it first. After each item, there are two very similar sentences, sentence (a) and sentence (b). Please judge, based on the reading you have just read, how each sentence sounds. Each of the sentences is followed by the following scale for you to rate each sentence.

**Table:** 

You cannot say it like this	I think it's bad, but I'm not sure	I don't think it is very bad, but I don't think it's the best way to say it	It's more or less OK	It's perfect to say it like that	I don't know if you can say it like this
1	2	3	4	5	0

Here is an example:

You always really liked churros with chocolate. When you were young, whenever you saw churros, you would tell your mother:

(a) I want to eat them. 1 2 3 4 ➄ 0
(b) Them I want to eat. 1 2 3 4 ➄ 0

The test also emphasized that any combination of numbers was possible [i.e., sentence (a) could be 5 and sentence (b) could be 1, or both of them could be 5, etc.]. Subjects were asked to do the test as quickly as possible, as we were only interested in their first intuitions.

### Results

#### General results

An Five-Way mixed repeated measures ANOVA was conducted in which verb type (unaccusative, unergative), word order (SV, VS), prototypicality (3 levels), information structure (focus, non-focus), and participant group (monolingual, bilingual) were entered as variables. Results revealed, first, main effects of verb type, *F*_(1, 38)_ = 5.29, *p* = 0.027, η^2^ = 0.122, word order, *F*_(1, 38)_ = 7.71, *p* = 0.008, η^2^ = 0.169 and prototypicality, *F*_(2, 76)_ = 21.25, *p* < 0.001, η^2^ = 0.359. Unergative sentences tended to receive higher scores (4.12, *SEM* = 0.076) than unaccusative sentences (4.03, *SEM* = 0.064); sentences with SV order received higher acceptability scores overall (4.20, *SEM* = 0.074) than those with VS order (3.95, *SEM* = 0.090); and sentences with prototypical and intermediate verbs received higher acceptability scores overall (prototypical 4.13, *SEM* = 0.075, intermediate 4.17, *SEM* = 0.073) than those with non-prototypical verbs (3.93, *SEM* = 0.066), pairwise comparisons *p* < 0.001.

**Figure 5:**
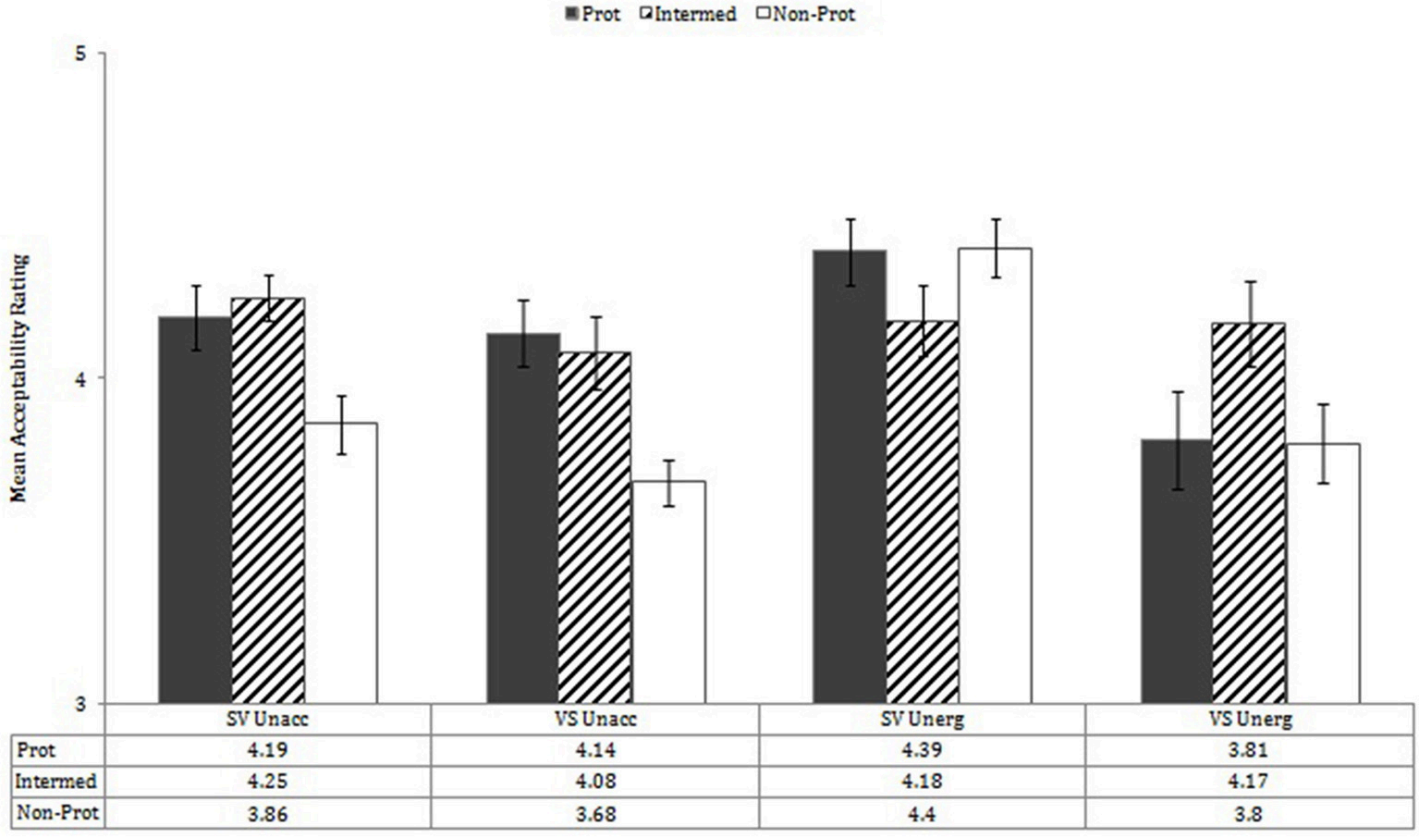
**Mean acceptability ratings by word order X verb type X prototypicality**. Error bars in all figures show SEM.

These main effects were modified, however, by interaction effects. First, there were interactions of Verb Type X Prototypicality, *F*_(2, 76)_ = 12.07 *p* < 0.001, η^2^ = 0.241; Verb Type X Word Order, *F*_(1, 38)_ = 8.06, *p* = 0.007, η^2^ = 0.175; Prototypicality X Word Order, *F*_(2, 76)_ = 4.98, *p* = 0.009, η^2^ = 0.116; and Verb Type X Prototypicality X Word Order, *F*_(2, 76)_ = 3.98, *p* = 0.023, η^2^ = 0.095. Performance by verb type, prototypicality, and word order is shown in Figure 5.

Follow-up analyses in which each verb type was analyzed separately via mixed-effect repeated measures (with word order (SV, VS), prototypicality (3 levels), information structure (focus, non-focus), and participant group (monolingual, bilingual) as variables) revealed that for unaccusatives, performance by word order was not significant, nor was performance by Word Order X Prototypicality; there was, however, a significant overall effect of prototypicality, *F*_(2, 76)_ = 40.29, *p* < 0.001, with sentences involving non-prototypical verbs judged less acceptable than those with prototypical or intermediate verbs, *p*s < 0.001.

For unergatives, in contrast, judgments varied by word order, *F*_(1, 38)_ = 10.12, *p* = 0.003, and by Word Order X Prototypicality, *F*_(2, 76)_ = 6.94, *p* = 0.002. For SV unergative sentences, prototypicality was significant, *F*_(2, 76)_ = 3.28, *p* = 0.043, but only in relation to significantly higher acceptability ratings of SV with non-prototypical verbs than with intermediate verbs (*p* = 0.029) (and near-significantly higher with prototypical than intermediate, *p* = 0.083). For VS unergative sentences, judgments showed a reverse pattern: Prototypicality was significant, *F*_(2, 76)_ = 5.90, *p* = 0.004, but VS sentences built on intermediate verbs received higher scores than those built on either prototypical or non-prototypical verbs, *p* = 0.008, *p* = 0.011, respectively.

Thus, for unaccusatives, word order in general did not affect performance (but see results concerning information structure, below), and sentences built on non-prototypical verbs were judged less acceptable than those in which prototypical and intermediate verbs were used. For unergatives, in contrast, SV sentences were in general judged acceptable (but less so for the intermediate verbs), and VS sentences were judged less acceptable, especially in cases in which prototypical and non-prototypical verbs occurred.

**Figure 6:**
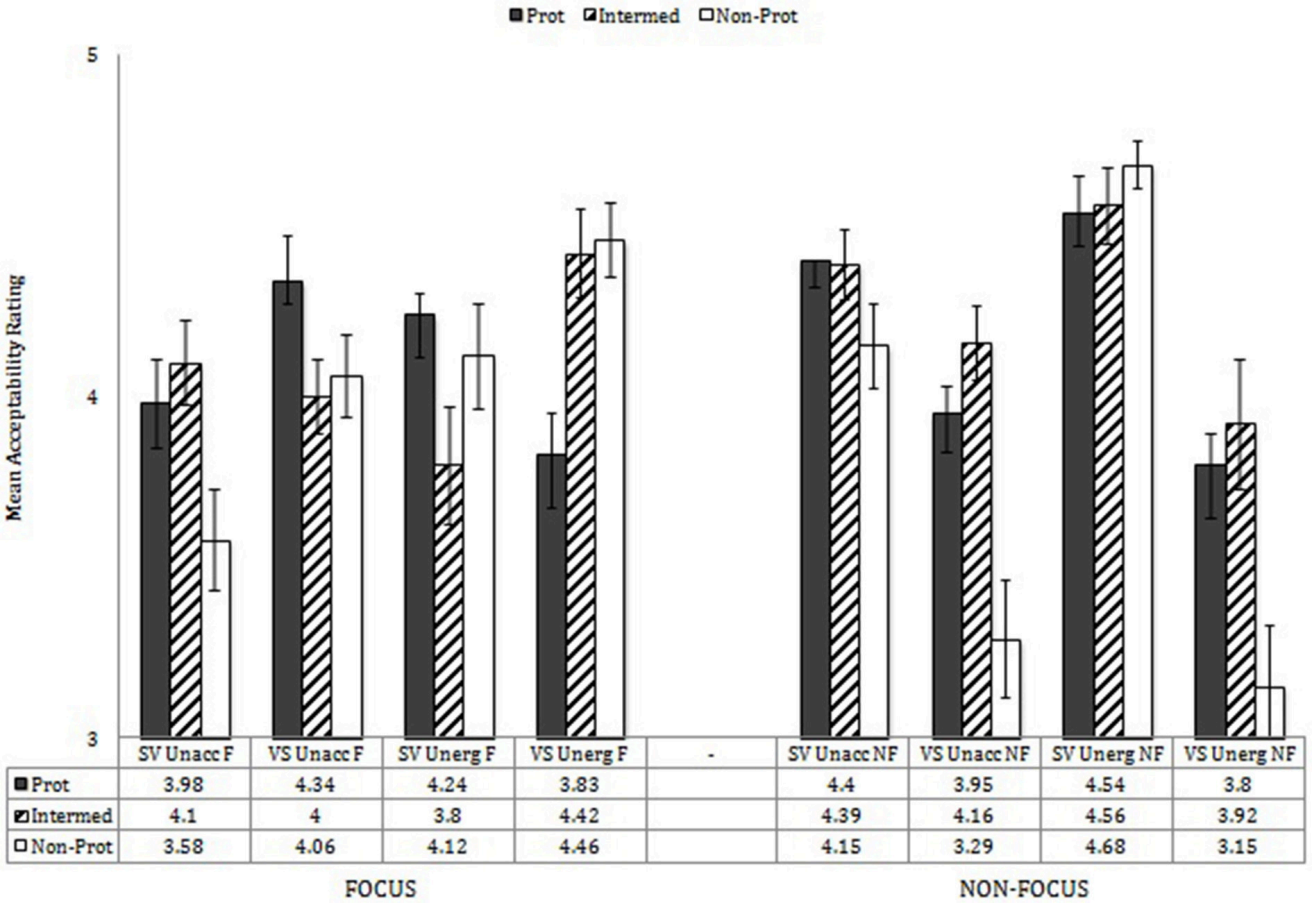
**Performance by verb type, prototypicality, word order, and information structure**.

The main analyses also showed significant interactions of Word Order X Information Structure, *F*_(1, 38)_ = 47.39, *p* < 0.001, η^2^ = 0.555; Prototypicality X Information Structure, *F*_(2, 76)_ = 13.72, *p* < 0.001, η^2^ = 0.265; Verb Type X Prototypicality X Information Structure, *F*_(2, 76)_ = 4.03, *p* = 0.022, η^2^ = 0.092; Prototypicality X Word Order X Information Structure, *F*_(2, 76)_ = 8.59, *p* < 0.001, η^2^ = 0.184; and Verb Type X Prototypicality X Word Order X Information Structure, *F*_(2, 76)_ = 6.53, *p* = 0.002, η^2^ = 0.147. Performance by verb type, prototypicality, word order, and information structure is shown in Figure 6. These effects were explored, first, by analysing each verb type separately.

For unaccusative verbs, there were interactions of Prototypicality X Information Structure, *F*_(2, 76)_ = 8.26, *p* = 0.001; Word Order X Information Structure, *F*_(1, 38)_ = 24.05, *p* < 0.001; and Prototypicality X Word Order X Information Structure, *F*_(2, 76)_ = 4.61, *p* = 0.013. Unaccusatives with SV word order showed significant differences in acceptability by prototypicality, *F*_(2, 76)_ = 12.35, *p* < 0.001: SV was more accepted with prototypical and intermediate verbs than with non-prototypical verbs, *p* = 0.001, *p* < 0.001, respectively. Unaccusatives with VS word order showed significant effects of prototypicality, *F*_(2, 76)_ = 14.54, *p* < 0.001, information structure, *F*_(1, 38)_ = 15.68, *p* < 0.001, and of Prototypicality X Information Structure, *F*_(2, 76)_ = 10.67, *p* < 0.001. In focus contexts, acceptability of unaccusatives with VS order showed significant effects by prototypicality, *F*_(2, 76)_ = 4.83, *p* = 0.011, with higher acceptability ratings with prototypical verbs than with either intermediate or non-prototypical verbs, *p* = 0.002, *p* = 0.035, respectively. In non-focus contexts, acceptability of unaccusatives with VS order differed across the three prototype levels, *F*_(2, 76)_ = 16.38, *p* < 0.001: prototypical less than intermediate *p* = 0.018; prototypical greater than non-prototypical *p* < 0.001; intermediate greater than non-prototypical *p* < 0.001. Interestingly, these results show higher acceptability ratings of VS in non-focus contexts with intermediate unaccusative verbs than with prototypical unaccusatives.

For unergative verbs, there was a main effect of word order, *F*_(1, 38)_ = 10.12, *p* = 0.003, and there were significant interactions of Prototypicality X Word Order, *F*_(2, 76)_ = 6.94, *p* = 0.002; Prototypicality X Information Structure, *F*_(2, 76)_ = 10.34, *p* < 0.001; Word Order X Information Structure, *F*_(1, 38)_ = 36.63, *p* < 0.001, and Prototypicality X Word Order X Information Structure, *F*_(2, 76)_ = 13.23, *p* < 0.001. When unergatives occurred with SV word order, effects of prototypicality, *F*_(2, 76)_ = 3.28, *p* = 0.043, informational structure, *F*_(1, 38)_ = 24.70, *p* < 0.001, and of Prototypicality X Informational Structure, *F*_(2, 76)_ = 3.28, *p* = 0.043, reveal that whereas in non-focus contexts, there was no difference by prototypicality (note that SV structures with unergatives in non-focus contexts received the highest acceptability ratings out of all groups), in focus contexts, prototypicality effects were evident, *F*_(2, 76)_ = 4.34, *p* = 0.016, with higher acceptability ratings in relation to prototypical and non-prototypical verbs than with intermediate verbs, *p* = 0.013, *p* = 0.048, respectively. When unergatives were used with VS word order, in focus contexts, significant effects of prototypicality, *F*_(2, 76)_ = 12.02, *p* < 0.001, indicate lower ratings with prototypical verbs than with intermediate or non-prototypical verbs, *p* = 0.001, *p* < 0.001, respectively. When unergatives occurred with VS order in non-focus contexts, significant effects of prototypicality, *F*_(2, 76)_ = 11.05, *p* < 0.001, indicate significantly lower ratings with non-prototypical verbs than with either prototypical or intermediate verbs, *p* < 0.001, *p* = 0.001, respectively.

These results indicate that in non-focus contexts, SV order is accepted (in fact, preferred) with all verb types. Further, in non-focus contexts, VS order is more accepted with prototypical and intermediate verbs of both types (unergative and unaccusative) than with non-prototypical verbs.

In focus contexts, the results are more complex: With unaccusative verbs, VS order is preferred for prototypical and non-prototypical verbs, but with intermediate verbs, SV and VS are equally accepted. With unergative verbs, VS is preferred with intermediate and non-prototypical verbs, but SV is preferred with prototypical verbs.

These overall results are sometimes consistent with word order predictions regarding unaccusative and unergative verbs, sometimes inconsistent. Consistent with predictions, all unergatives in non-focus contexts are accepted with SV word order, and prototypical and intermediate unaccusative verbs are accepted with VS order. And in focus contexts, prototypical unaccusatives are accepted with VS order; however, prototypical unergatives are disfavored with VS order. Inconsistent with predictions, however, are the following: In non-focus contexts all types of unaccusative verbs are judged acceptable with SV order, and in focus contexts, prototypical unergative verbs are judged acceptable, in fact preferred, with SV word order.

#### Participant groups

Returning to the main analyses, let us now examine effects concerning participant groups. There was a near-significant effect of Verb Type X Word Order X Participant Group, *F*_(1, 38)_ = 3.44, *p* = 0.071, η^2^ = 0.083, a significant interaction of Word Order X Information Structure X Participant Group, *F*_(1, 38)_ = 9.47, *p* = 0.004, η^2^ = 0.199, and a near-significant interaction of Prototypicality X Word Order X Information Structure X Participant Group, *F*_(2, 76)_ = 2.82, *p* = 0.066, η^2^ = 0.069.

**Figure 7:**
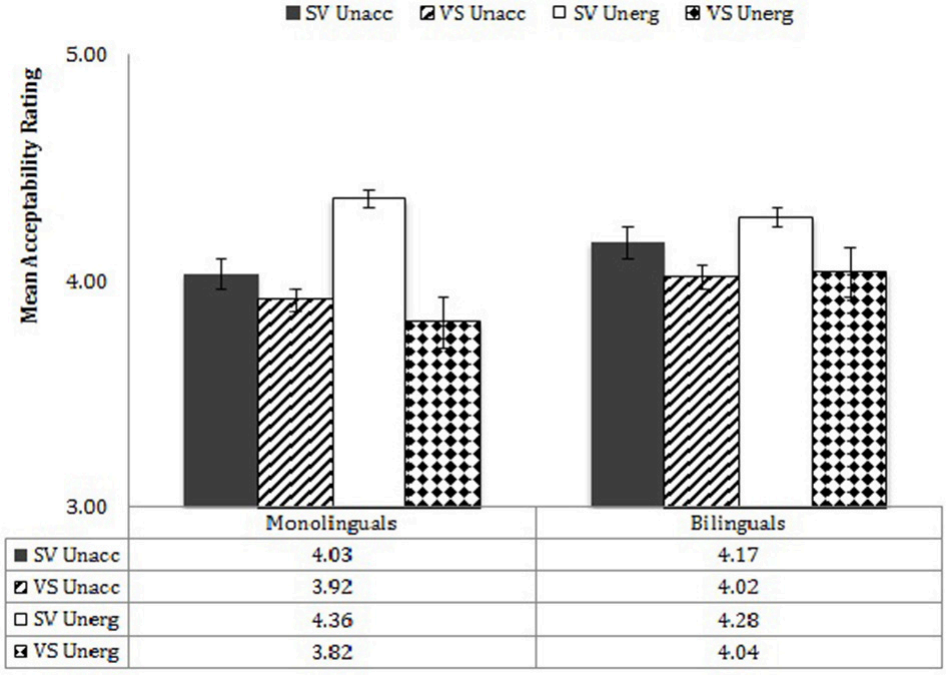
**Verb type X WO X participant group**.

To explore these interactions, performance of the monolinguals and bilinguals was analyzed separately in mixed effects analyses with verb type (unaccusative, unergative), word order (SV, VS), prototypicality (3 levels), and information structure (focus, non-focus) as variables. Performance by each group by verb type and word order is shown in Figure 7. ANOVAs for the two separate participant groups showed that the bilinguals showed no main effect of either verb type or word order, nor an interaction of Verb Type X Word Order, whereas monolinguals showed significant effects for all three—verb type, *F*_(1, 19)_ = 5.70, *p* = 0.03, η^2^ = 0.231, word order, *F*_(1, 19)_ = 6.55, *p* = 0.019, η^2^ = 0.256, Verb Type X Word Order, *F*_(1, 19)_ = 5.60, *p* = 0.007, η^2^ = 0.329. This means that, while the monolinguals distinguished the privileges of occurrence of the two types of verbs relative to word order, the bilinguals did not make any distinction between the two verb types and accepted both word orders about equally (but see below with regard to non-focus contexts).

**Figure 8:**
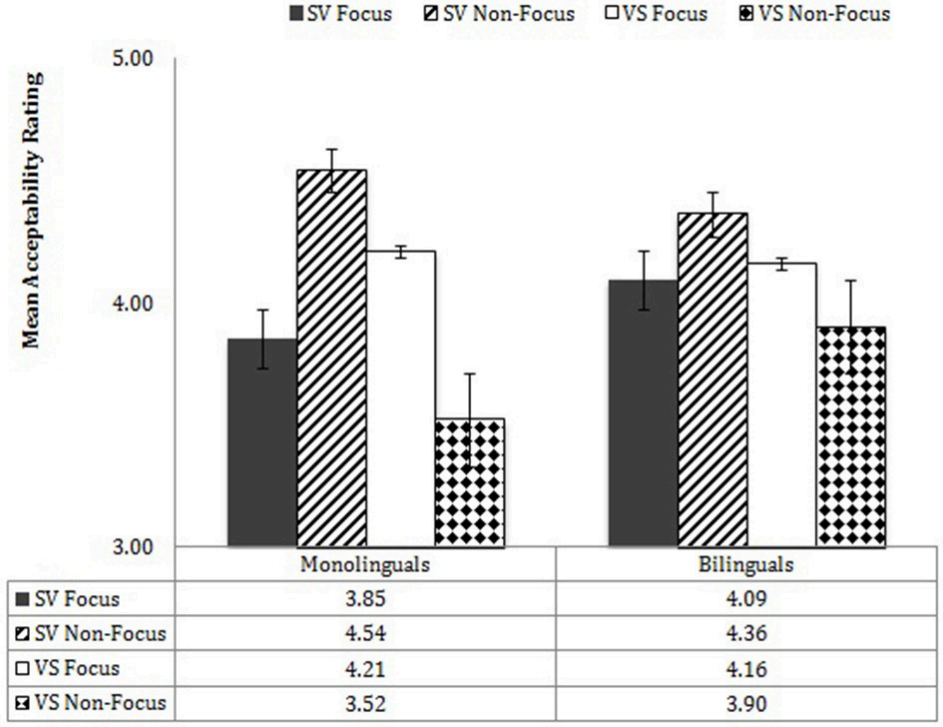
**Word order X information structure X participant group**.

Monolinguals' and bilinguals' performance by Word Order X Information Structure is shown in Figure 8. Bilinguals showed a significant interaction of Word Order X Information Structure, *F*_(1, 19)_ = 13.44, *p* = 0.002, η^2^ = 0.419, as did the monolinguals, *F*_(1, 19)_ = 33.96, *p* < 0.001, η^2^ = 0.641. In the case of the bilinguals, in the Focus contexts, there was no significant difference in judgments for SV vs. VS word order [*F*_(1, 19)_ = 0.22, *p* = 0.643]; in the Non-Focus contexts, there was a significantly higher acceptance of SV order (4.36) than VS order (3.90). In the case of the monolinguals, in the Focus contexts, there was a near-significant preference for VS order (4.21) over SV order (3.85), *F*_(1, 19)_ = 3.47, *p* = 0.078; in the Non-Focus contexts, there was a dramatic preference for SV order (4.54) over VS order (3.52), *F*_(1, 19)_ = 44.95, *p* < 0.001. Thus, the greatest differences between the two participant groups are that monolinguals showed a preference for VS order in Focus contexts, whereas bilinguals did not differentiate orders in those contexts, and the monolinguals showed a more dramatic categorical choice of SV over VS in non-focus contexts than the bilinguals, even though the latter also showed a significant difference in the acceptability of SV over VS in non-focus contexts.

**Figure 9:**
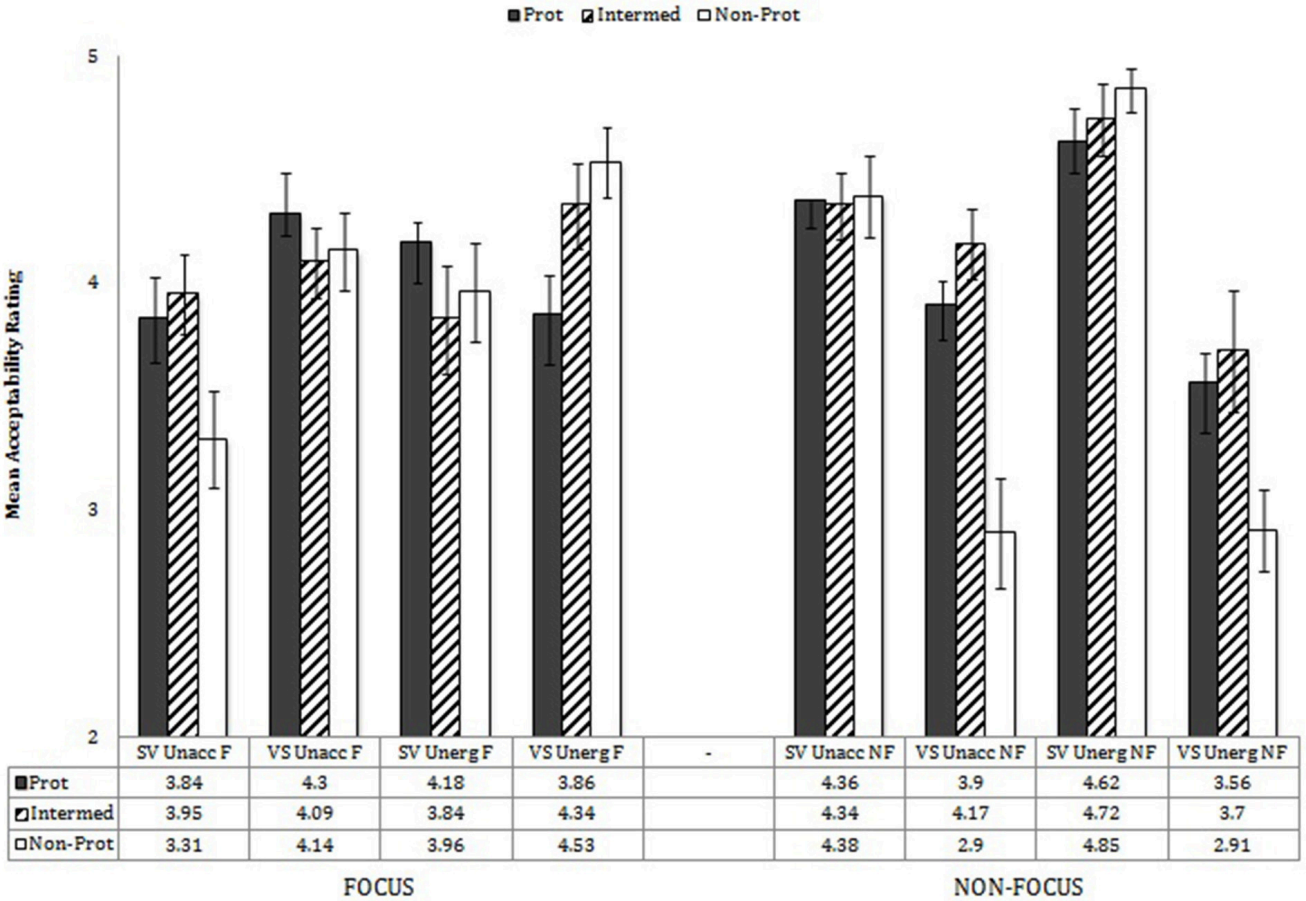
**Monolinguals' performance by verb type X word order X focus X prototypicality**.

**Figure 10:**
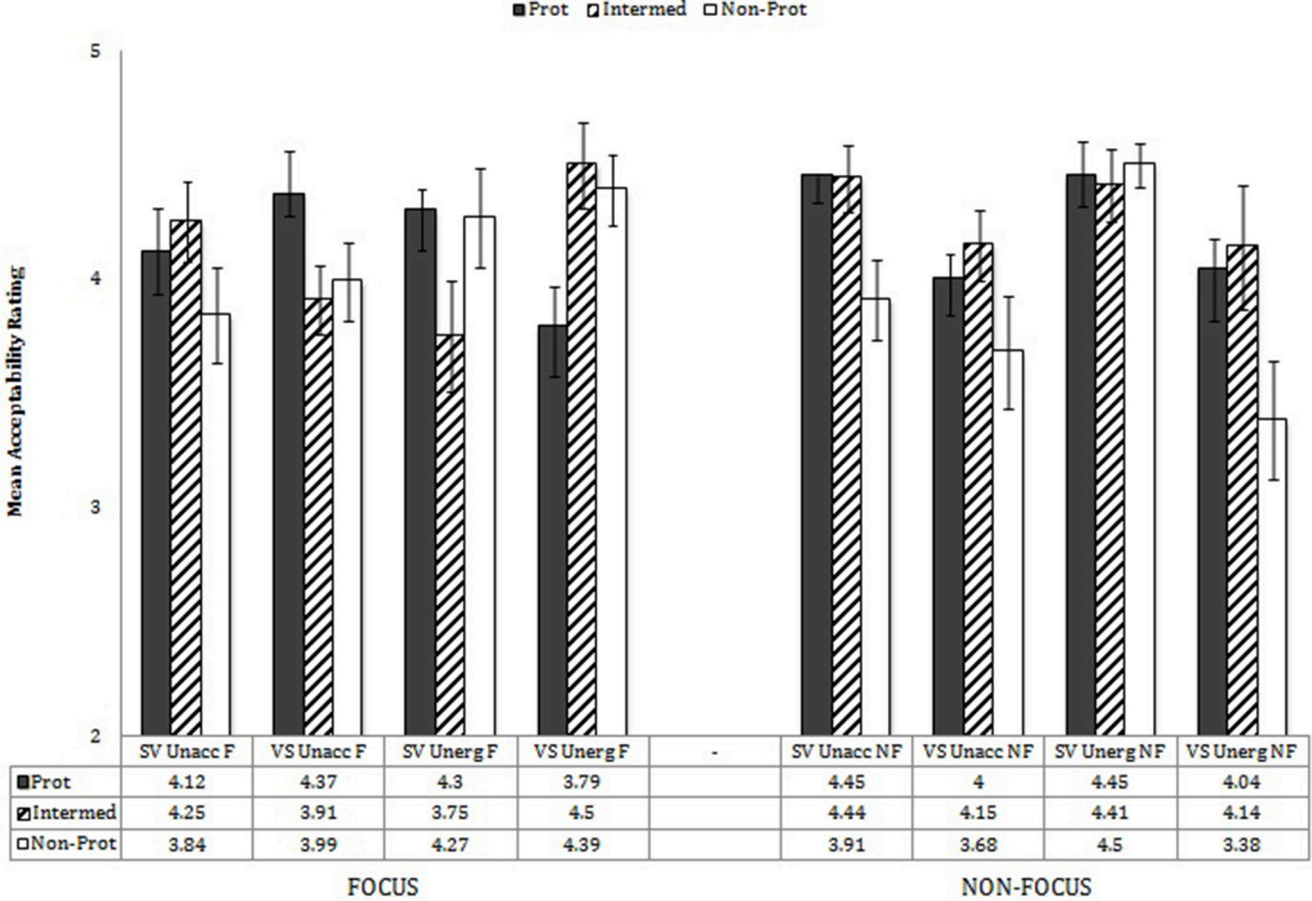
**Bilinguals' performance by verb type X word order X focus X prototypicality**.

To explore the near-significant interaction of Prototypicality X Word Order X Information Structure X Participant Group, the two participant groups' performance was compared for the three prototypical levels in a Two-Way mixed ANOVA (with prototype level and participant group as variables) for each verb type in each condition—focus/non-focus and SV/VS. Performance of the monolinguals by Prototypicality X Word Order X Information Structure is shown in Figure 9 and of the bilinguals in Figure 10. Comparisons showed that the only significant differences in performance patterns for the two participant groups relative to prototypicality, word order, and information structure occurred with unaccusative verbs in the non-focus conditions, for both SV and VS order. With SV order for unaccusatives in the non-focus condition, there was an interaction between Prototypicality X Participant Group, *F*_(2, 76)_ = 3.23, *p* = 0.045: While the monolinguals treated all prototypical levels equivalently here (accepting SV for all prototypicality levels), the bilinguals showed lower acceptability ratings for the constructions with the non-prototypical verbs here than with prototypical or intermediate verbs, *p* = 0.007, *p* = 0.006, respectively. With VS order for unaccusatives in the non-focus condition, an interaction between Prototypicality X Participant Group, *F*_(2, 76)_ = 3.68, *p* = 0.03, revealed that while the bilinguals showed no significant difference in performance by prototypicality, the monolinguals found the constructions with non-prototypical verbs here to be less acceptable than those with the prototypical and intermediate verbs, *p's < 0.001*, and those with the prototypical verbs marginally less acceptable than those with intermediate verbs, *p* = 0.07. Thus, in general, the patterns of responses relative to the three prototypicality levels within each condition were similar for monolinguals and bilinguals. The exceptions were the following: (1) In the case of unaccusatives in non-focus contexts, monolinguals found SV order uniformly acceptable, while bilinguals tended to accept SV with non-prototypical verbs less than with prototypical and intermediate verbs. (2) In non-focus contexts, bilinguals treated VS orders as equally acceptable with unaccusatives at all prototypicality levels, but monolinguals tended to disallow VS with non-prototypical unaccusative verbs.

## Discussion

These results overall indicate the following:

With regard to the general findings:

(1) The acceptability ratings for unaccusatives and unergatives were consistent with some of the predictions:
  (a) In non-focus contexts, SV was the preferred order for all types of unergative verbs,
  (b) In non-focus contexts, VS was judged slightly more acceptable with (prototypical and intermediate) unaccusative verbs than with (prototypical and intermediate) unergative verbs, and
  (c) In focus contexts, VS order was preferred with (prototypical and non-prototypical) unaccusative verbs and with (intermediate and non-prototypical) unergative verbs.

(2) The acceptability ratings for unaccusatives and unergatives were not entirely as expected, however. In particular,
  (a) In non-focus contexts, SV order was the preferred order for both verb types, including unaccusatives,
  (b) SV order was accepted for most verbs even in focus contexts (with the exception of non-prototypical unaccusative verbs and possibly intermediate unergative verbs),
  (c) VS order was accepted in non-focus contexts more for prototypical and intermediate unergatives than for non-prototypical unergatives (similar to what was found for the unaccusatives), and
  (d) VS order in focus contexts was treated as fairly unacceptable for prototypical unergative verbs.

With regard to the participant groups:

(3) First, the bilinguals did not distinguish unaccusative verbs from unergative verbs (unlike the monolinguals);
(4) The bilinguals did not distinguish SV from VS order in focus contexts (unlike the monolinguals); and
(5) The bilinguals' favoring of SV over VS order in non-focus contexts was less dramatic than the monolinguals' preference for SV in this context.
(6) Finally, at the same time, the bilinguals' performance relative to prototypicality levels of the particular verbs in particular contexts was similar on the whole to that of the monolinguals, except in two minor cases.

These results inform the questions at hand. First, with regard to the question of whether the bilinguals have come to a higher command of the verb type distinctions (internal interface) than of the operation of focus on syntactic structure (external interface), these data provide a resounding “no.” Bilinguals did not on the whole differentiate the two verb types, and they only differentiated focus from non-focus contexts in that, whereas they accepted SV and VS orders equally in focus contexts, they favored SV order in non-focus contexts.

At the same time, however, it is clear that the bilinguals' performance was not random—their performance relative to particular verbs, as judged by the prototypicality effects, was similar to that of the monolinguals, but just at a less categorical or less extreme level.

Our findings with regard to the pervasiveness of use of SV with both unaccusative and unergative verbs, and the acceptance of VS even with unergative verbs, challenge the position that these verbs fall into a dichotomy. In order to explore these results further and to gain a better understanding of the findings, for each participant group, the performance on each verb was plotted and the verbs placed on a continuum.

**Figure 11:**
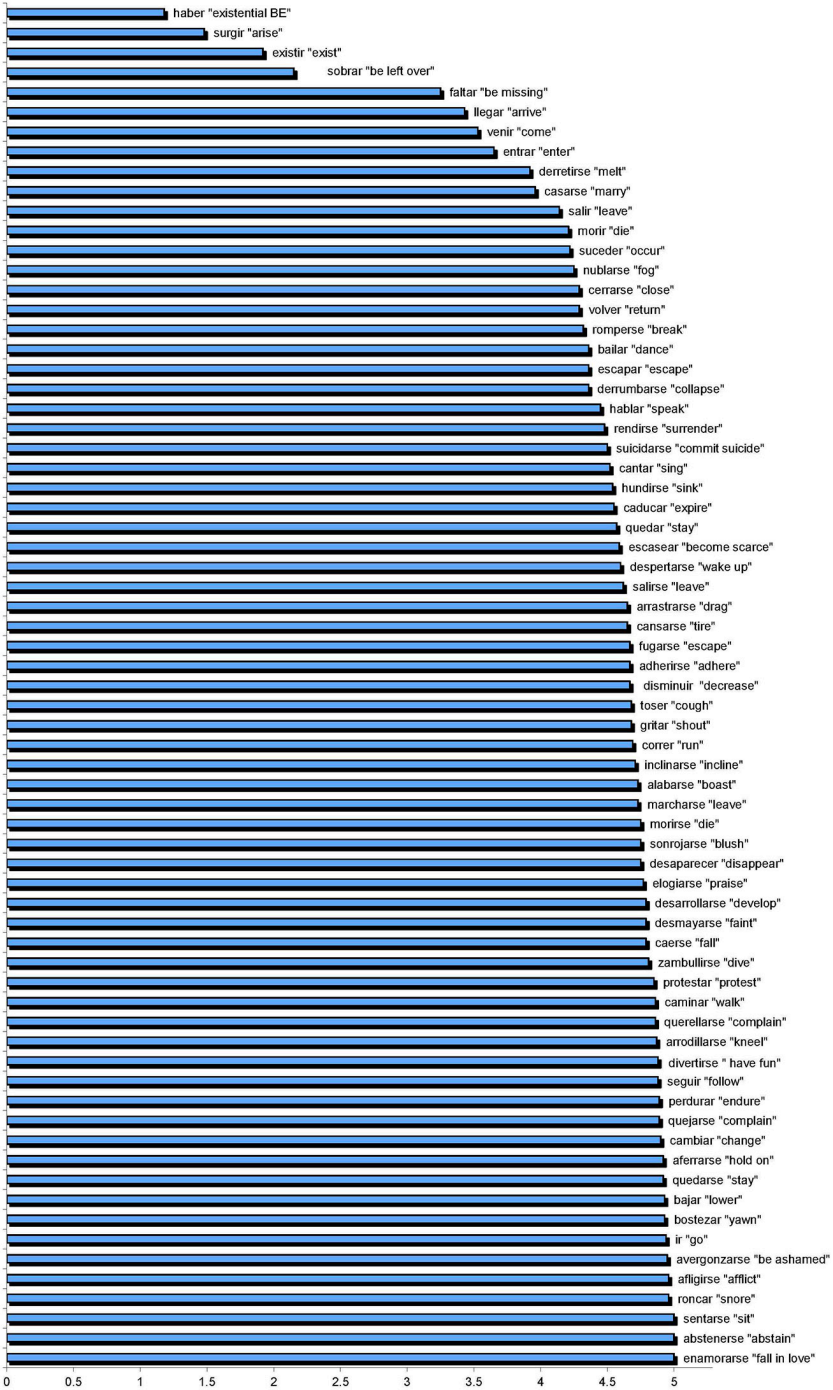
**Judgments, individual verbs in SV non-focus contexts**.

The acceptability scores for each verb when it occurred with SV order in non-focus contexts are shown in Figure 11 for the monolingual participants. Contrary to Lozano's claims that SV and VS do not alternate freely in native speaker grammars and that Spanish speakers treat the constructions categorically, our data show that there is a continuum, rather than a dichotomy.

**Figure 12:**
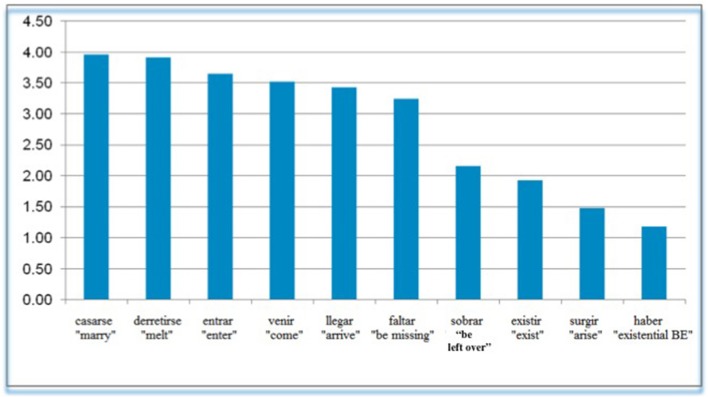
**True unaccusatives- lowest ratings for SV in non focus condition**.

The “true” unaccusatives should be those that were rated low when they occurred with SV order in non-focused contexts. The ten verbs with lowest acceptability ratings were those shown in Figure 12.

(Low SV in Non Focus Contexts).

**Figure 13:**
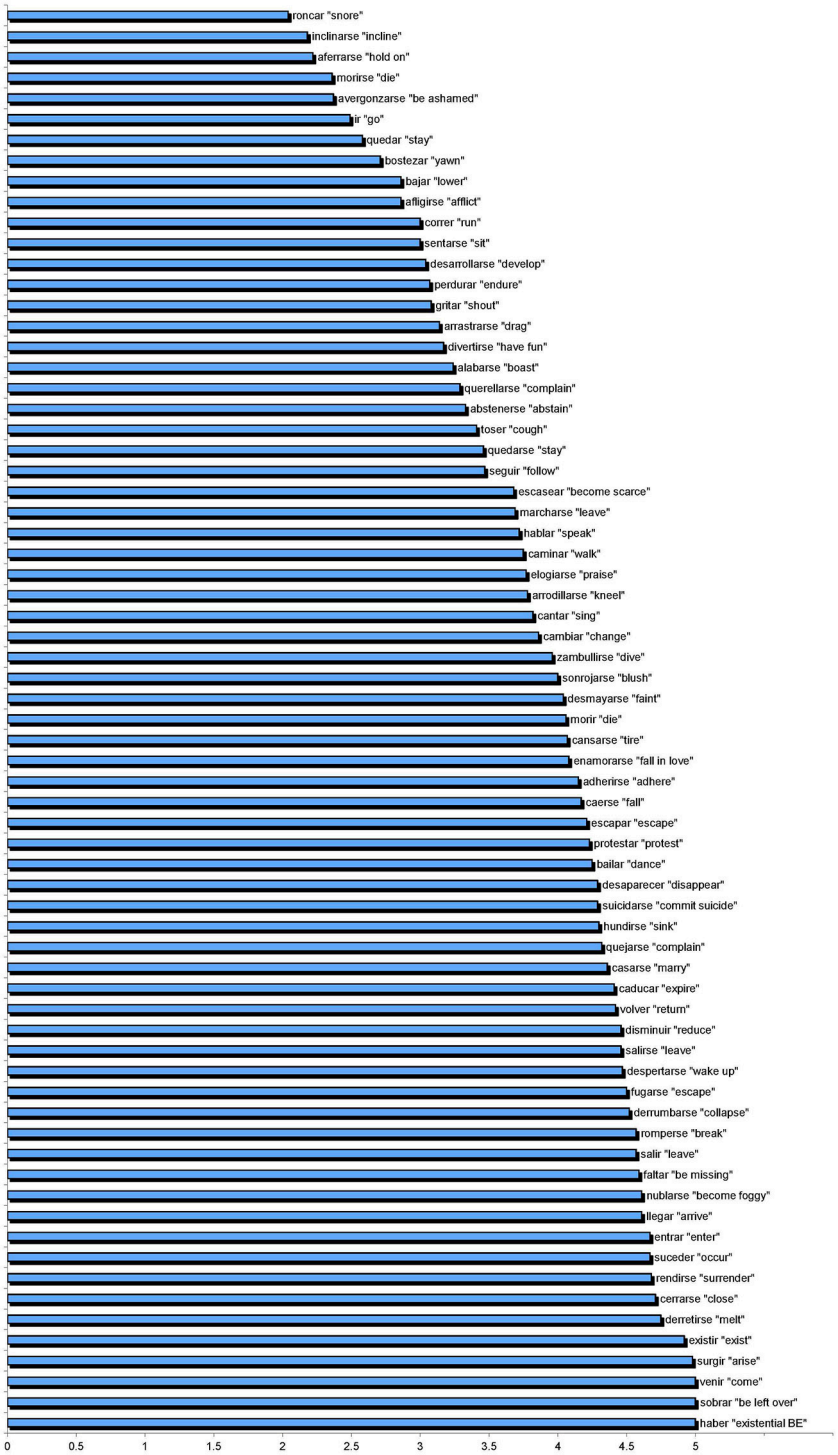
**Judgments, individual verbs in VS non-focus contexts**.

The acceptability scores for each verb when it occurred with VS order in a non-focus context are shown in Figure 13.

It is interesting not only that the data reveal no clear-cut across-the-board binary distinction between unergatives and unaccusatives, but also that the data also do not follow Sorace's hierarchical semantic continuum. These results are in line with those reported in de Prada Pérez and Pascual y Cabo (2012). Such findings merit further exploration. What factors are influencing speakers' judgments of these constructs? One possibility has to do with exposure to the particular individual verbs in context. Perhaps speakers have more marked judgments in relation to verbs they experience more frequently. To examine this possibility, we extracted the frequency of occurrence of each verb (in all its forms) from SUBTLEX-ESP (Cuetos et al., 2011) (out of 41,577,673 words), and we conducted correlational analyses of these frequencies relative to performance in each of the major contexts—SV Focus (SVF), SV non-Focus (SVnF), VS Focus (VSF), and VS non-Focus (VSnF). These analyses were conducted, first, with verbs of all types together, and then with the unergative and unaccusative verbs separately, for both the Monolinguals and the Bilinguals.

**Figure 14:**
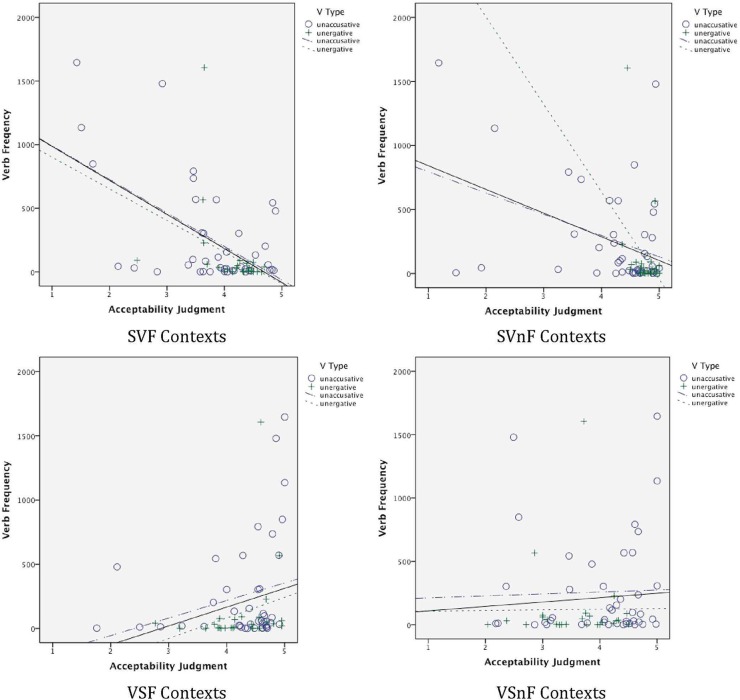
**Monolingual judgments**.

For the Monolinguals, first, for all verbs together (*N* = 69), the speakers' judgments showed a high correlation between judgments in the SVF, SVnF, and VSF contexts with the frequency of the verb. In SVF and SVnF settings, the correlation was negative, *r* = −0.544, *p* < 0.001, and *r* = −0.384, *p* = 0.001, respectively, indicating that the more frequent the verb, the less they accepted the SV order in both F and nF contexts. In VSF contexts, the correlation was positive, *r* = 0.260, *p* = 0.031, indicating that the more frequent the verb, the greater the acceptance of the VS order in F contexts. For unaccusative verbs alone (*N* = 43), negative correlations still held for SVF and SVnF, at *r* = −0.577, *p* < 0.001, and *r* = −0.385, *p* = 0.010, respectively, but the positive correlation in relation to VSF did not reach significance (*r* = 0.254, *p* = 0.100). For unergative verbs alone (*N* = 26), none of these reached significance, although for SVF and SVnF contexts, the correlations were near-significant, *r* = −0.354, *p* = 0.076, and *r* = −0.362, *p* = 0.076, respectively. Scatter plots showing the Monolinguals' judgments relative to verb frequency in each type of context are shown in Figure 14. (The slopes for all verbs together are shown with solid lines; those for the unaccusatives and unergatives separately with dotted lines as indicated.)

**Figure 15:**
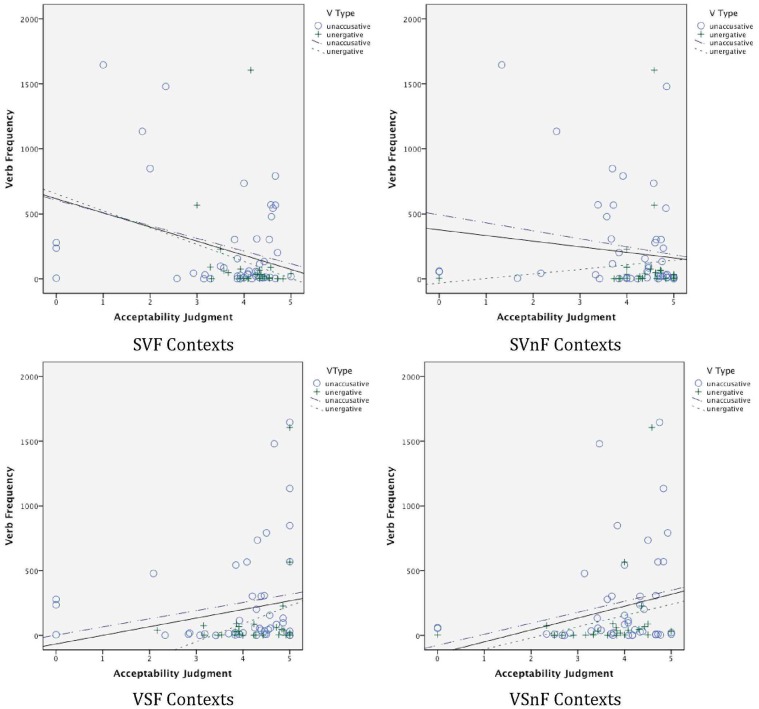
**Bilingual judgments**.

For the Bilinguals, for all verbs together (*N* = 72), there was a negative correlation between judgments in SVF contexts and frequency, *r* = −0.318, *p* = 0.006, again indicating that the more frequent the verb, the lower the judgments were for the verb in SV order in F contexts. There was also a positive correlation of frequency of the verb with acceptance of VS order in nF contexts, *r* = 0.260, *p* = 0.027. This latter result is surprising, as it indicates that the frequency with which a verb is heard correlates with higher acceptance of VS order even in non-Focus contexts. Neither of these correlations held for unergative verbs alone (*N* = 26), but for unaccusative verbs (*N* = 46), the negative correlation held in relation to SVF contexts, *r* = −0.320, *p* = 0.030. Scatter plots of the Bilinguals' judgments relative to verb frequency are shown for each context in Figure 15.

These correlations suggest that judgments are highly influenced by exposure to the particular verb in the given construction. For the Monolinguals, the more frequent the verb, the less the acceptance of SV order, in both F and nF contexts (the latter especially in the case of unaccusative verbs). Similarly, the more frequent the verb, the more they accept VS order in F contexts. The Bilinguals show a similar effect in SVF contexts—the more frequent the verb, the less they accept this order in F contexts. The fact that the Bilinguals pattern like the Monolinguals in these SVF contexts is further support for the conclusion drawn above that Bilinguals have in fact acquired features of the grammar related to the interaction of the discourse and syntax. Furthermore, all of these effects are in line with grammatical accounts regarding SV and VS order in F contexts—with speakers disfavoring SV order in F contexts for presumably well-ingrained verbs.

The similarity of verb patterns for unaccusative and unergative verbs, however, argues against a strong dichotomy between the two verb types, in line with what we have argued above. The effect showing that the Bilinguals are more likely to be more accepting of VSnF structures for more frequent verbs is difficult to explain, however. The effect is weak, but is deserving, as are the other correlations discovered, of more targeted research in future work.

## Conclusions

This study has explored syntactic, pragmatic, and lexical influences on adherence to SV and VS orders in native and fluent L2 speakers of Spanish who were long-standing functional bilinguals. The primary issue addressed has been the hypothesis that bilinguals have no difficulty in acquiring within-module grammatical elements and only encounter difficulties in relation to external interface phenomena. The data presented here do not support this position. Here, first, the bilinguals treated unaccusative and unergative verbs in identical fashion; this indicates that they have not gained a native-like command of this feature. In contrast, long-term L2 bilinguals did differentiate between focus and non-focus, in that they preferred SV order in non-focus contexts. Hence, our findings do not support the hypothesis that internal interfaces are acquired more easily than external interfaces (contra accepted wisdom- Sorace, 2005; Sorace and Filiaci, 2006; White, 2006).

A further result of this study concerns the classification of verbs themselves. Unlike previous empirical studies (Hertel, 2003; Lozano, 2006a,b) and contra the theoretical literature (Contreras, 1978; Suñer, 1982 and Zubizarreta, 1998), the data revealed no clear-cut across-the-board binary distinction between unergatives and unaccusatives (but see Domínguez and Arche, 2014). Neither monolinguals nor bilinguals differentiated the two types of verbs in non-focus situations. However, monolinguals paid attention to verb type in the focus situation in that they preferred VS for unaccusatives. Long-term L2 bilinguals did not differentiate between the two types of verbs, treating unaccusatives and unergatives equally even in focus contexts. The follow-up analyses showed strong correlations between speakers' judgment performance with the frequency of the verbs. These findings suggest that any account of the use of these verbs in these contexts will need to take into account a usage-based perspective. That is, it appears that performance on each verb is determined at least in part by speakers' relative experience with the verbs in question, rather than strict verb categories based either on syntax or semantics.

Two additional findings of the study are worth noting. First, bilinguals' judgments were less categorical overall than monolinguals'. That is, monolinguals were more likely to give extreme “yes” or “no” judgments, while bilinguals gave more intermediate judgments (cf. lower confidence ratings by bilinguals in their judgments compared to monolinguals in Sagarra and Herschensohn (2013). Second, individual verbs do not necessarily behave as predicted under standard definitions of unaccusatives and unergatives.

The data presented here do not lend support to a split intransitivity dichotomy. Rather, they support a continuum. This continuum, however, does not seem to fit Sorace's criteria defined primarily by aspectual notions (telicity/atelicity), and secondarily by the degree of agentivity of the verb. The functional bilinguals' performance differed from the performance of monolinguals in their lack of differentiation of verb types. And contrary to what is predicted according to the interface hypothesis, bilinguals differentiated between focus and non-focus situations.

Our results challenge the second version of the interface hypothesis (Sorace, 2011) by postulating that external interfaces (syntax-discourse) are not necessarily more difficult than internal interfaces (lexicon-syntax). They also provide new empirical evidence on the Unaccusative Hierarchy (semantic subclasses of unaccusatives and unergatives) for native Spanish, which does not work in the same way as proposed for Italian. In addition, the results of this study shed new light on word order alternations (SV/VS) which, although previously studied, needed to be investigated in more detail in long-standing functional bilinguals if we wanted a better understanding of this phenomenon in end-state grammars.

If confirmed and broadened through further research using a wider range of methodologies with learners at different levels of proficiency, the findings of this study have fundamental implications for our understanding of the interface system in L2 learners and for our general understanding of the grammar of unaccusative and unergative verbs.

### Conflict of interest statement

The authors declare that the research was conducted in the absence of any commercial or financial relationships that could be construed as a potential conflict of interest.

## Footnotes

^1^Functional bilingualism is one's ability to use and produce both languages across “an encyclopedia of everyday events” (Baker, 1993:13).

^2^Lozano's instrument can be downloaded from the IRIS database (http://www.iris-database.org)

## References

[B1] Baker C. (1993). Foundations of Bilingual Education and Bilingualism. Clevedon: Multilingual Matters.

[B2] Baker M. (1983). Objects, themes and lexical rules in Italian, in Papers in Lexical-Functional Grammar,” eds Levin L. Rappaport Hovav M. Zaenen A. (Bloomington, IN: Indiana University Linguistics Club), 1–46.

[B3] Beck M. L. (1998). L2 acquisition and obligatory head movement: English-speaking learners of German and the local impairment hypothesis. Stud. Second Lang. Acquis. 20, 311–348.

[B4] Belletti A. (2001). ‘Inversion’ as focalization, in Inversion in Romance and the Theory of Universal Grammar, eds Hulk A. Pollock J. Y. (Oxford: Oxford University Press), 60–90.

[B5] Belletti A. (2004). Aspects of the low IP area, in The Structure of IP and CP. The cartography of Syntactic Structures, Vol. 2, ed Rizzi L. (Oxford: Oxford University Press), 16–51.

[B6] Belletti A. Leonini C. (2004). Subject inversion in L2 Italian, in Eurosla Yearkbook 4, eds Foster-Cohen S. Sharwood Smith M. Sorace A. Ota M. (Amsterdam: John Benjamins Publishing Company), 95–118.

[B7] Burzio L. (1986). Italian Syntax: A Government and Binding Approach. Dordrecht: Reidel.

[B8] Camacho J. (1999). From SOV to SVO: the grammar of interlanguage word order. Second Lang. Res. 15, 115–132.

[B9] Chomsky N. (1981). Lectures on Government and Binding. Dordrecht: Foris.

[B10] Chomsky N. (1995). The Minimalist Program. Cambridge, MA: The MIT Press.

[B11] Chomsky N. (2005). Three factors in language design. Linguist. Inq. 36, 1–22 10.1162/0024389052993655

[B12] Contreras H. (1978). El orden de Palabras en Español. Madrid: Cátedra.

[B13] Cowart W. (1997). Experimental Syntax: Applying Objective Methods to Sentence Judgments. Thousand Oaks, CA: SAGE.

[B14] Cuetos F. Glez-Nosti M. Barbon A. Brysbaert M. (2011). SUBTLEX-ESP: Spanish word frequencies based on film subtitles. Psicologica 32, 133–143.

[B15] De Miguel E. (1993). Construcciones ergativas e inversión en la lengua y la inter-lengua españolas, in La lingüística y el Análisis de Los Sistemas No Nativos, ed Liceras J. (Ottawa, ON: Dovehouse), 178–195.

[B16] de Pra da Pérez A. Pascual y Cabo D. (2012). Interface heritage speech across proficiencies: unaccusativity, focus, and subject position in Spanish, in Selected Proceedings of the 14th Hispanic Linguistics Symposium, eds Geeslin K. Díaz-Campos M. (Somerville, MA: Cascadilla Proceedings Project), 308–318.

[B17] Domínguez L. Arche M. J. (2014). Subject inversion in non-native Spanish. Lingua 145, 243–265 10.1016/j.lingua.2014.04.004

[B18] Dowty D. R. (1991). Thematic proto-roles and argument selection. Language 67, 547–619.

[B19] Friedmann N. Taranto G. Shapiro L. P. Swinney D. (2008). The vase fell (the vase): the online processing of unaccusatives. Linguist. Inq. 39, 355–377. 10.1162/ling.2008.39.3.355 22822348 PMC3399662

[B20] Hatcher A. (1956). Theme and underlying question: two studies of Spanish word order. Word 12, 3.

[B21] Hawkins R. (2000). Persistent selective fossilization in second language acquisition and the optimal design of the language faculty. Essex Res. Rep. Linguist. 34 75–90.

[B22] Hertel T. J. (2003). Lexical and discourse factors in the second language acquisition of Spanish word order. Second Lang. Res. 19, 273–304 10.1191/0267658303sr224oa

[B23] Levin B. Rappaport Hovav M. (1995). Unaccusativity at the Syntax-Lexical Semantics Interface. Cambridge, MA: MIT Press.

[B24] Lozano C. (2003). Universal Grammar and Focus Constraints: The Acquisition of Pronouns and Word Order in Non-Native Spanish. PhD dissertation. University of Essex.

[B25] Lozano C. (2006a). Focus and split intransitivity: the acquisition of word order alternations in non-native Spanish. Second Lang. Res. 22, 1–43 10.1191/0267658306sr264oa

[B26] Lozano C. (2006b). The development of the syntax-discourse interface: Greek learners of Spanish, in The Acquisition of Syntax in Romance Languages, eds Torrens V. Escobar L. (Amsterdam: John Benjamins), 371–399.

[B27] Lozano C. (2009). The Acquisition of Syntax and Discourse: Pronominals and Word Order in English and Greek Learners of Non-Native Spanish. Saarbrüken: VDM Verlag.

[B28] Montrul S. (2001). Causatives and transitivity in L2 English. Lang. Learn. 51, 51–106 10.1111/1467-9922.00148

[B29] Montrul S. (2004). Subject and object expression in Spanish heritage speakers: a case of morphosyntactic convergence. Bilingualism Lang. Cogn. 7, 125–142 10.1017/S1366728904001464

[B29a] Montrul S. (2005). On knowledge and development of unaccusativity in Spanish L2 acquisition. Linguistics 43, 1153–1190 10.1515/ling.2005.43.6.1153

[B30] Nava E. (2007). Word order in bilingual Spanish: convergence and intonation strategy, in Selected Proceedings of the Third Workshop on Spanish Sociolinguistics, eds Holmquist J. Lorenzino A. Sayahi L. (Somerville, MA: Cascadilla Proceedings Project), 129–139.

[B31] Ocampo F. (1990). The pragmatics of word order in constructions with a verb and a subject. Hisp. Linguist. 4, 87–127.

[B32] Oshita H. (2001). The unaccusative trap in second language acquisition. Stud. Second Lang. Acquis. 23, 279–304 10.1017/S0272263101002078

[B33] Perlmutter D. M. (1978). Impersonal passives and the unaccusativity hypothesis, in Proceedings of the Fourth Annual Meeting of the Berkeley Linguistic Society (Berkeley: Berkeley Linguistic Society; University of California), 157–189.

[B34] Perlmutter D. M. Postal P. M. (1984). The I-advancement exclusiveness law, in Studies in Relational Grammar 2, eds Perlmutter D. M. Rosen C. (Chicago, IL: University of Chicago Press), 81–125.

[B35] Pinto M. (1999). Information focus: between core and periphery, in Semantic Issues in Romance Syntax, eds Treviño E. Lema J. (Amsterdam: John Benjamins), 179–191.

[B36] Rappaport Hovav M. Levin B. (2001). An event structure account of English resultatives. Language 77, 766–797 10.1353/lan.2001.0221

[B37] Rosen C. (1984). The interface between semantic roles and initial grammatical relations, in Studies in Relational Grammar eds Perlmutter D. Rosen C. (Chicago, IL: University of Chicago Press), 38–77.

[B37a] Sagarra N. Herschensohn J. (2013). Processing of gender and number agreement in late Spanish bilinguals. Int. J. Bilingualism 17, 607–627 10.1177/1367006912453810

[B38] Schmerling S. (1976). Aspects of English Sentence Stress. Austin, TX: University of Texas Press.

[B39] Selkirk E. (1984). Phonology and Syntax: The Relation Between Sound and Structure. Cambridge, MA: MIT Press.

[B40] Sorace A. (1993a). Incomplete vs. divergent representations of unaccusativity in non-native grammars of Italian. Second Lang. Res. 9, 22–47.

[B41] Sorace A. (1993b). Unaccusativity and auxiliary choice in non-native grammars of Italian and French: asymmetries and predicable indeterminacy. J. Fr. Lang. Stud. 3, 71–93.

[B42] Sorace A. (1995). Acquiring linking rules and argument structures in a second language: the unaccusative/unergative distinction, in The Current State of Interlanguage, eds Eubank L. Selinker L. Sharwood M. S. (Amsterdam: Benjamins), 153–175.

[B43] Sorace A. (2000). Gradients in auxiliary selection with intransitive verbs. Language 76, 859–890 10.2307/417202

[B44] Sorace A. (2005). Syntactic optionality at interfaces, in Syntax and Variation: Reconciling the Biological and the Social, eds Cornips L. Corrigan K. (Amsterdam: John Benjamins), 46–111.

[B46] Sorace A. (2011). Pinning down the concept of “interface” in bilingualism. Linguist. Approaches Bilingualism 1, 1–33 10.1075/lab.1.1.01sor

[B47] Sorace A. Filiaci F. (2006). Anaphora resolution in near-native speakers of Italian. Second Lang. Res. 22, 339–368 10.1191/0267658306sr271oa

[B48] Sorace A. Shomura Y. (2001). Lexical constraints on the acquisition of split intransitivity: evidence from L2 Japanese. Stud. Second Lang. Acquis. 23, 247–278 10.1017/S0272263101002066

[B49] Suñer M. (1982). Syntax and Semantics of Spanish Presentational Sentence-types. Washington, DC: Georgetown University Press.

[B50] Tsimpli I. Sorace A. (2006). Differentiating interfaces: L2 performance in syntax-semantics and syntax-discourse phenomena, in Proceedings of the 30th Annual Boston University Conference on Language Development (Somerville, MA: Cascadilla Press), 653–664.

[B51] van Hout A. (1996). Event Semantics of Verb Frame Alternations: A Case Study of Dutch and its Acquisition. Doctoral dissertation, Tilburg University.

[B52] van Hout A. Randall J. Weissenborn J. (1992). Acquiring the unaccusative/unergative distinction, in The Acquisition of Dutch Amsterdam Series in Child Language Development 1, eds Verrips M. Wijnen F. (Amsterdam: University of Amsterdam), 79–120.

[B53] van Valin R. (1999). Generalized semantic roles and the syntax-semantics interface, in Empirical Issues in Formal Syntax and Semantics 2, eds Corblin F. Dobrovie-Sorin C. Marandin J.-M. (The Hague: Thesus), 373–389.

[B54] White L. (2006). Interfaces and L2 knowledge: the Spanish connection, in Plenary address presented at the Hispanic and Luso-Brazilian Linguistics Symposium (London: The University of Western Ontario).

[B55] White L. (2009). Language acquisition at the interfaces: some hardy perennials and new varieties, in Mind-Context Divide Conference (Iowa City: University of Iowa).

[B56] Yip V. (1995). Interlanguage and Learnability: From Chinese to English. Amsterdam: Benjamins.

[B57] Zubizarreta M. L. (1998). Prosody, Focus, and Word Order. Cambridge, MA: MIT Press.

